# Constitutive activation of EGFR is associated with tumor progression and plays a prominent role in malignant phenotype of chondrosarcoma

**DOI:** 10.18632/oncotarget.26899

**Published:** 2019-05-07

**Authors:** Jun Qin, Irfan Shaukat, Didier Mainard, Patrick Netter, Lydia Barré, Mohamed Ouzzine

**Affiliations:** ^1^ UMR7365 Centre National de la Recherche Scientifique (CNRS), Université de Lorraine, Biopôle, Faculty of Medicine, Nancy 54505, France

**Keywords:** chondrosarcoma, EGF/EGFR signaling, biomarker, tyrosine kinase inhibitor, cell death

## Abstract

Chondrosarcoma is a highly agressive cancer with currently no effective therapies when unresectable or metastasized, thus the outcome remains poor. High-grade chordrosarcomas are resistant to conventional chemotherapy and radiotherapy and surgical resection remains the only treatment for the majority of chondrosarcomas. Constitutive activation of receptor tyrosine kinases has been shown to be important for malignant transformation and tumour proliferation. Here, we investigated the activation status of EGFR in chondrosarcoma tumor biopsies and cell lines. We found that EGFR is activated in grade II and grade III chondrosarcoma tumors but not in grade I tumors, suggesting a role in tumor progression. Interestingly, we showed that EGFR is activated through an autocrine loop and that inhibition of the EGFR by the TKI, tyrphostin AG1478 or EGFR neutralizing antibodies strongly reduced activation of oncogenic ERK1/2 and mTOR/AKT downstream pathways. Importantly, inhibition of EGFR profoundly reduces cell proliferation and migration, inhibits the expression of MMP13 and MMP3 and enhances cell death. Taken together, these data support the blocking of EGFR as new potential treatment for high-grade chondrosarcoma tumors.

## INTRODUCTION

Targeted therapy was recently recognized as promising strategy for cancer treatment [1]. Constitutive activation of receptor tyrosine kinases (RTKs) has been shown to be important for malignant transformation and tumour proliferation. Recently, there has been increased focus on developing anti-cancer therapies aimed at controlling key pathways governing oncogenesis and aggressive clinical features of tumor cells by inhibiting RTKs. Several small molecule inhibitors such as tyrosine-kinase inhibtors (TKIs) and antibodies are being clinically developed to target RTKs [2]. Examples of RTK inhibitors include imatinib, sunitinib and regorafenib in gastrointestinal stromal tumors [3, 4] and pazopanib in non-adipocytic soft tissue sarcomas [5]. Beside small molecule inhibitors, several antibodies are being clinically developed to target RTKs such as trastuzumab which is used to target the extracellular domain of the HER2 protein in HER2-positive breast cancer patients and has been shown to increase survival at early and late stages of breast cancer [6], and cetuximab used to target the EGFR-ligand binding in the treatment of patients with metastatic colorectal cancer [7].

Chondrosarcoma is a rare cancer that accounts for about 20% of bone tumors which show hyaline cartilage differentiation and display diverse histopathology and behaviour [8]. They are associated with high metastatic potential and poor prognosis and new therapeutic approaches are urgently needed. Indeed, chondrosarcomas are poorly responsive to radiation and conventional chemotherapy [9], thereby the clinical management of chondrosarcomas remains a challenging problem. Due to the lack of an effective adjuvant therapy, surgical resection remains the only treatment for the majority of chondrosarcomas. Despite previous evidences for activation of PDGFRB in chondrosarcomas [10], targeting the PDGF pathway by the PDGFR TKI imatinib mesylate failed to show any significant activity or clinical success [11], suggesting that other pathways may be involved in chondrosarcoma pathogenesis. Therefore, identifying chondrosarcoma’s key signaling pathways involved in tumor development and survival is an important issue towards rational development of efficient therapies. Activation of RTK signaling often leads to cell transformation, which is observed in a wide variety of malignancies. This, results in the activation of MAP kinase and PI3K/AKT pathways leading to increase in cell proliferation, survival, invasion and metastasis. The epidermal growth factor receptor (EGFR) is widely up-regulated in solid tumors and mediates many characteristics of malignant phenotype, including proliferation, tumor cell motility and cell survival marking it as a good target for therapeutic intervention [12–14]. Therapies targeting EGFR using TKIs and antibodies directed against EGFR-ligand binding site have provided remarkable responses in human non-small cell lung cancer [15, 16] and metastatic colorectal cancer [7, 17], respectively.

Here, we investigated the activation status of EGFR in chondrosarcoma tumors and studied the effect of inhibition of EGFR in chondrosarcoma cell lines using specific TKI inhibitor and neutralizing antibodies. We found that EGFR is activated in grade II and grade III chondrosarcoma tumors but not in grade I tumors. Most important, we found that EGFR is activated through an autocrine loop and showed that inhibition of EGFR profoundly reduced the activation of both ERK1/2 and AKT/mTOR signaling and decreased cell proliferation and migration of chondrosarcoma cells. In addition, our results showed that inhibition of EGFR produced a cell cycle arrest at G0/G1 phase and induced apoptosis of chondrosarcoma cells. Moreover, we found that expression of MMP-13 and MMP-3 proteinases involved in the degradation of cartilage extracellular matrix (ECM), were down regulated following the inhibition of EGFR. This study reveals that activation of EGFR is a key event that drives tumorigenesis in chondrosarcoma and suggests that inhibition of EGFR by a selective TKI or neutralizing antibodies may constitute a potential treatment for chondrosarcoma tumors.

## RESULTS

### EGFR is activated in chondrosarcoma tumors

**Figure 1:**
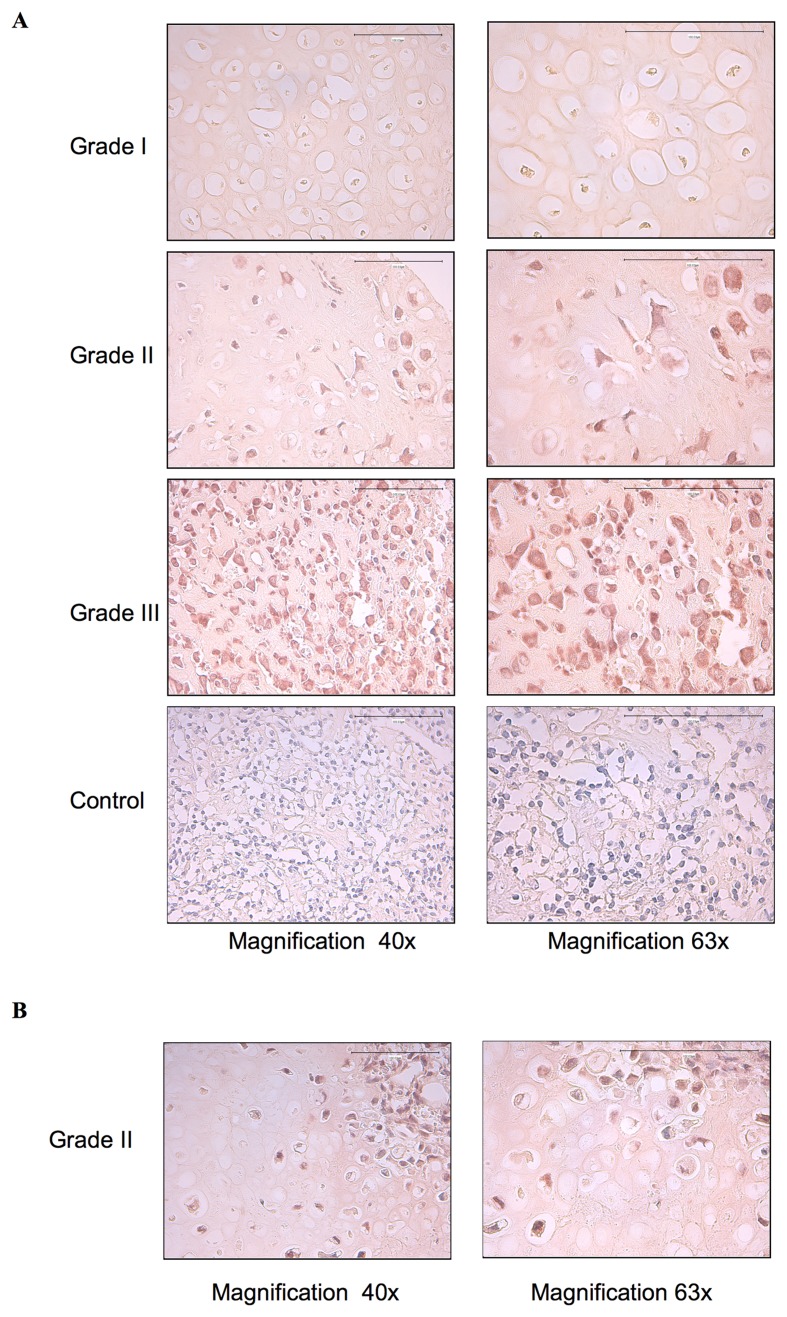
EGFR is constitutively activated in high-grade chondrosarcoma tumor biopsies. (**A**) Twenty-seven chondrosarcoma tumor biopsies of grade I (*n* = 14), grade II (*n* = 6) and Grade III (*n* = 7) were probed with anti-p-EGFR antibodies. Representative images are shown at magnification (×40) and (×63). (**B**) Grade II chondrosarcoma tumor biopsy showing the phospho-EGFR staining in cluster of cells in the biopsy. Anti-p-EGFR antibodies were missed in the control. Brown color indicates positive cells.

**Figure 2:**
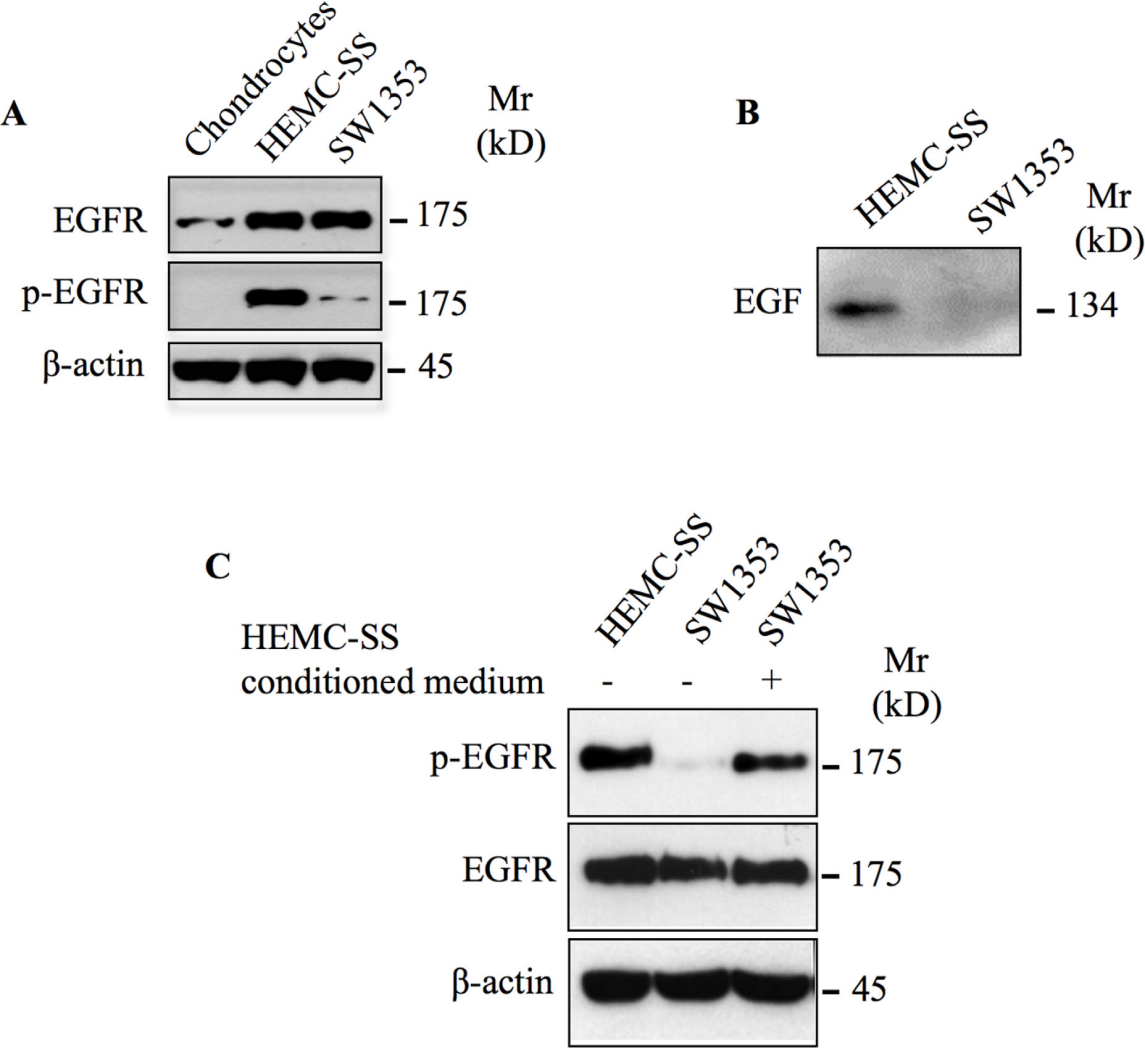
EGFR is overexpressed and constitutively activated in chondrosarcoma cells. (**A**) Western blot analysis of EGFR and p-EGFR in primary chondrocytes and in chondrosarcoma cell lines HEMC-SS and SW1353. (**B**) Detection of EGF in conditioned medium of chondrosarcoma cell lines HEMC-SS and SW1353. (**C**) Western blot analysis of EGFR and p-EGFR in HEMC-SS, SW1353 and SW1353 stimulated for 1 h with conditioned medium from HEMC-SS cells. β-actin was used as a loading control. Data are representative of three independent experiments (*n* = 3).

Aberrant activation and deregulated expression of EGFR has been found to be important for cancer cell proliferation, survival and invasion as well as resistance to chemotherapy [18, 19]. To determine whether EGFR was activated in chondrosarcoma tumors, we analyzed the phosphorylation status of this receptor in twenty-seven chondrosarcoma tumor biopsies of different grade i.e., fourteen grade I, six grade II and seven grade III by immunohistochemistry using specific phospho-EGFR antibodies. We found that EGFR is activated in grade III and in grade II, whereas it is not activated in grade I tumors (Figure 1A). Indeed, all grade III and grade II chondrosarcoma tumor biopsies analyzed were stained positively for phosphorylated EGFR, however no staining was observed in grade I tumors (Figure 1A). Unexpectedly, phospho-EGFR staining was restricted to clusters of cells in in 30% of grade II chondrosarcoma tumor biopsies (Figure 1B), suggesting that activation of EGFR is an event that occurs during chondrosarcoma tumor progression. To further validate the EGFR immunohistochemistry data, we analyzed the expression and phosphorylation of EGFR in two human-derived chondrosarcoma cell lines, HEMC-SS and SW1353, and in human primary chondrocytes by immunoblot. The results revealed that EGFR is strongly expressed in chondrosarcoma cell lines compared to primary chondrocytes (Figure 2A). Analysis of the phosphorylation status of EGFR indicated that the receptor is strongly activated in chondrosarcoma cell line HEMC-SS but weakly activated in SW1353. In contrast, no detectable activation of EGFR is observed in primary chondrocytes (Figure 2A). These results indicate that EGFR expression is up-regulated in chondrosarcoma cells compared to primary chondrocytes, suggesting misregulation of EGFR gene expression in chondrosarcoma tumors. Given that constitutive activation of EGFR is high in HEMC-SS chondrosarcoma cells compared to SW1353 cells, while protein express level of the receptor is similar in both cells, we hypothesized that constitutive activation of the receptor in HEMC-SS chondrosarcoma cells may be due to an EGF/EGFR autocrine loop. To test this hypothesis, we examined whether SW1353 and HEMC-SS chondrosarcoma cells produce EGF. For these purposes, conditioned medium from SW1353 and HEMC-SS cells were collected after 24 h of growth and expression of EGF was analyzed by western blot. The results clearly show that HEMC-SS chondrosarcoma cells produce significant amount of EGF, whereas SW1353 cells did not produce detectable amount of this growth factor (Figure 2B), therefore suggesting an autocrine loop in HEMC-SS chondrosarcoma. Given that SW1353 chondrosarcoma cells express high amount of EGFR, we examined whether incubation of SW1353 cells with conditioned medium from HEMC-SS cells induces the phosphorylation of the receptor. As shown in Figure 2C, EGFR receptor was strongly activated following stimulation of SW1353 cells with conditioned medium from HEMC-SS cells, indicating that SW1353 cells express high amount of functional EGFR but do not express EGFR ligand.

### Constitutive EGFR signaling mediates aberrant activation of ERK1/2 and AKT in chondrosarcoma

**Figure 3:**
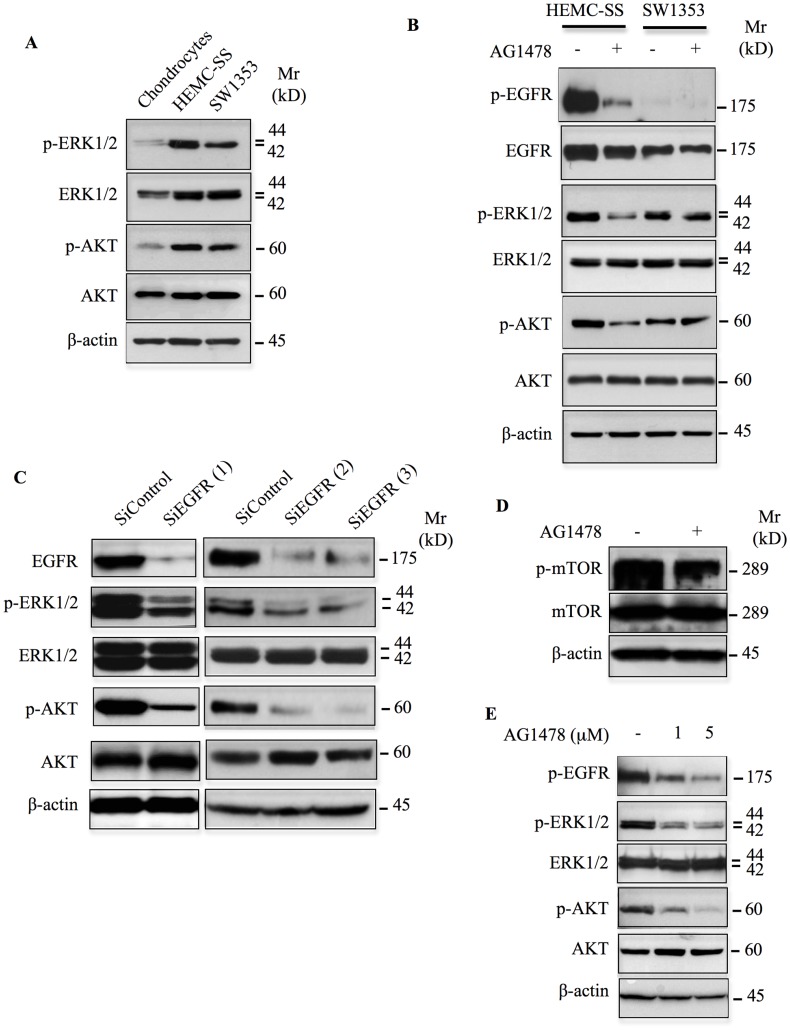
Inhibition or silencing EGFR down regulates ERK1/2 and AKT/mTOR signaling pathways. (**A**) Western blot analysis of the activation status of ERK1/2 and AKT signaling pathways in chondrosarcoma cells and primary chondrocytes. (**B**) Analysis of the effect of AG1478 (1 μM) on the phosphorylation of EGFR and on the activation of ERK1/2 and AKT downstream signaling pathways in chondrosarcoma cells HEMC-SS and SW1353. Control cells were treated with DMSO (vehicle). (**C**) Western blot analysis of the expression of EGFR and the phosphorylation status of ERK1/2 and AKT in chondrosarcoma HEMC-SS cells treated with siRNA specific to EGFR (siEGFR) or siRNA control (siControl). (**D**) Western blot analysis of the effect of AG1478 (1 μM) on the activation of mTOR in HEMC-SS chondrosarcoma cells. Control cells were treated with DMSO (vehicle). (**E**) Analysis of the effect of AG1478 at 1 μM and 5 μM on the phosphorylation of EGFR and on the activation of ERK1/2 and AKT downstream signaling pathways in chondrosarcoma cells HEMC-SS cultured in 3D in alginate beads. β-actin was used as a loading control. Data are representative of three independent experiments (*n* = 3).

We showed above that EGFR is activated in chondrosarcoma cells. Given that EGFR activation triggers known oncogenic signals such as ERK1/2 and AKT and promote malignant phenotype, we analyzed the activation status of these pathways in HEMC-SS and SW1353 chondrosarcoma cells, and in human primary chondrocytes. Western blot analysis showed that both ERK1/2 and AKT signaling pathways were strongly activated in chondrosarcoma cells compared to chondrocytes (Figure 3A). To determine whether constitutive activation of ERK1/2 and AKT is dependent on EGFR activation, we examined the effect of inhibition of EGFR on the activation status of these signaling pathways. To this end, we used tyrphostin AG1478, a highly potent and selective inhibitor of EGFR. We first tested whether AG1478 inhibits the phosphorylation of EGFR receptor in chondrosarcoma cells. As shown in Figure 3B, treatment of chondrosarcoma cells with AG1478 strongly inhibits the phosphorylation of EGFR. Importantly, inhibition of EGFR severely reduced the activation of both ERK1/2 and AKT signaling pathways in HEMC-SS chondrosarcoma but not in SW1353 cells (Figure 3B), indicating that activation of ERK1/2 and AKT signaling in HEMC-SS chondrosarcoma cells depends on EGFR activation, whereas it is not the case in SW1353.

To further confirm the role of EGFR in the activation of downstream pathways, we investigated the effect of EGFR knockdown on the phosphorylation status of ERK1/2 and AKT in HEMC-SS cells. Silencing of EGFR by siRNA efficiently reduced its expression (Figure 3C) and strongly decreased the phosphorylation of both ERK1/2 and AKT (Figure 3C), confirming the key role of EGFR in the activation of downstream signaling pathways in HEMC-SS chondrosarcoma cells. Altogether, these data suggest that aberrant activation of ERK1/2 and AKT signaling pathways in chondrosarcoma cells is driven by constitutive EGFR activation.

It is well known that mTOR acts both upstream and downstream of AKT and plays an important role in the regulation of cell growth and proliferation. To determine whether mTOR is activated in HEMC-SS chondrosarcoma cells and whether this is associated with EGFR activation, we analyzed mTOR phosphorylation status in the absence and presence of the EGFR inhibitor, AG1478. As shown in Figure 3D, mTOR is strongly phosphorylated in chondrosarcoma cells. Interestingly, inhibition of EGFR following treatment with AG1478 markedly decreased the activation of mTOR (Figure 3D), therefore suggesting that EGFR mediates the activation AKT/mTOR signaling pathways in HEMC-SS chondrosarcoma cells.

### Chondrosarcoma cells cultured in 3D are sensitive to anti-EGFR treatment

*In vitro* three-dimensional (3D) models, are known to permit endogenous ECM deposition, cell-cell matrix interactions and cell-cell contact in all directions, allowing cell responses that more closely mimic events occurring *in vivo* during cancer formation and progression. Therefore, it is important to evaluate the effect of the EGFR inhibitor, AG1478 on chondrosarcoma cells cultured in 3D system. To this end, HEMC-SS chondrosarcoma cells were embedded in alginate beads and grown for one week before treatment with AG1478 or DMSO (control) for 24 h. Treatment of HEMC-SS chondrosarcoma cells cultured in 3D with AG1478 at 1 μM and 5 μM inhibits the phosphorylation of EGFR and reduced the activation of both ERK1/2 and AKT signaling pathways, however the inhibition is more pronounced when 5 μM of AG1478 were used (Figure 3E). These data suggest that AG1478 is able to inhibit the activation of EFGR pathway in HEMC-SS tumoral cells that grow in a 3D environment.

### Anti-EGFR neutralizing antibodies inhibit constitutive activation of downstream signaling pathways

**Figure 4:**
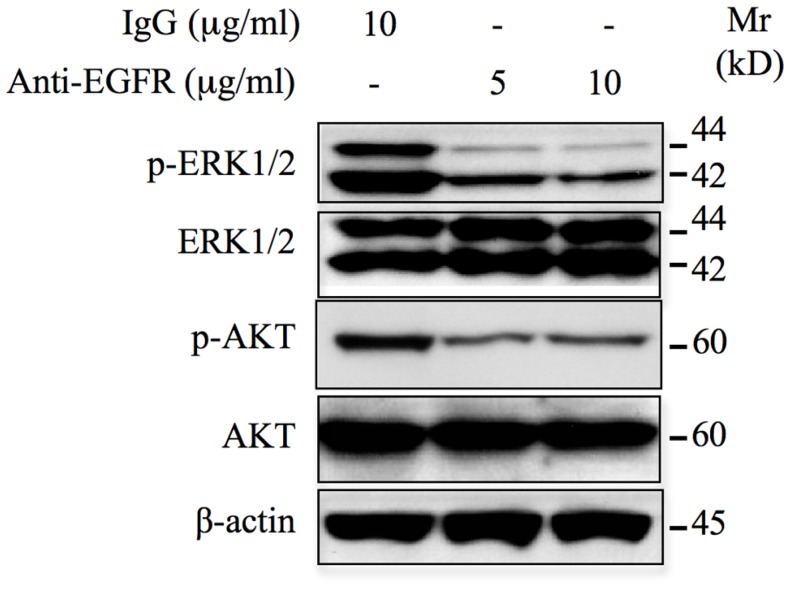
Monoclonal anti-EGFR neutralizing antibodies inhibit ERK1/2 and AKT signaling pathways. Western blot analysis of the effect of EGFR neutralizing antibodies (5 μg/ml and 10 μg/ml) on the activation of ERK1/2 and AKT pathways in chondrosarcoma HEMC-SS cells. For control, cells were incubated with 10 μg/ml of IgG. β-actin was used as a loading control. Data are representative of three independent experiments (*n* = 3).

The use of monoclonal antibodies for cancer therapy has been successful and their clinical development is growing. Monoclonal antibodies targeting EGFR-ligand binding domain have been used for the treatment of colorectal cancer [20], however their use in chondrosarcoma is not reported yet. To determine whether such strategy could be considered in the case of chondrosarcoma, we examined whether anti-EGFR monoclonal antibodies are effective in preventing the aberrant activation of ERK1/2 and AKT pathways. For this purpose, HEMC-SS chondrosarcoma cells were incubated with a monoclonal anti-EGFR neutralizing antibody that reacts with external domain of EGFR and the phosphorylation status of ERK1/2 and AKT were monitored by western blot. As shown in Figure 4, ERK1/2 and AKT signaling were down regulated in the presence of the anti-EGFR antibody. Indeed, strong inhibition of ERK1/2 and AKT phosphorylation was observed at concentration of 5 μg/ml anti-EGFR neutralizing antibody. No inhibition of these signaling pathways was seeing when incubation is carried out with anti-IgG antibodies. This observation indicates that anti-EGFR antibodies are able to efficiently block the activation of oncogenic signals, ERK1/2 and AKT in chondrosarcoma cells.

### Inhibition of EGFR decreases proliferation and migration of chondrosarcoma cells

**Figure 5:**
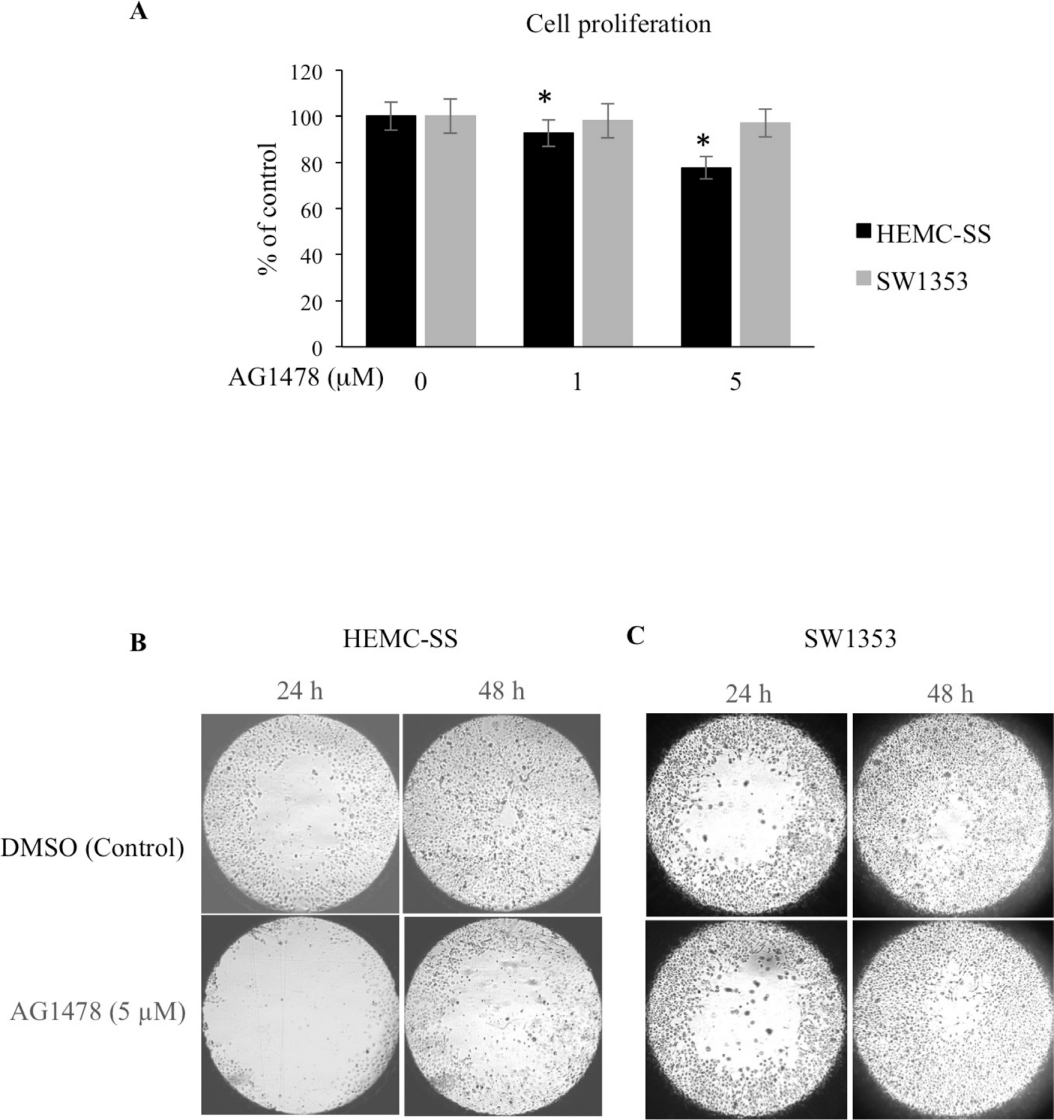
AG1478 reduces proliferation and migration of chondrosarcoma cells. (**A**) HEMC-SS chondrosarcoma cells were treated with DMSO (Control) or with 1 μM or 5 μM of AG1478 and cell proliferation was analyzed by Click-iT^®^ EdU as described in Material and Methods. Bar graph shows cell proliferation expressed as percentage relative to control cells. Data are expressed as mean (*n* = 3) ± SD. ^*^P < 0.05 versus control. (**B**) and (**C**) HEMC-SS and SW1353 chondrosarcoma cells cultured in 96 well-plates were allowed to attach overnight, then stoppers were removed creating a central cell-free zone. Culture medium containing AG1478 (5 μM) or DMSO (control) was added and cell migration to central zone was monitored at 24 h and 48 h.

We showed above that inhibition of EGFR strongly decreases ERK1/2 signaling. Given the important role of this pathway in cell proliferation, it is likely that inhibition of EGFR may affect cell growth. To test this hypothesis, we assessed the effect of inhibition of EGFR on the proliferation of human chondrosarcoma cells. As shown in Figure 5A, a dose-dependent inhibition in cell growth of chondrosarcoma cells HEMC-SS was observed following treatment with the EGFR inhibitor AG1478. Up to 30% inhibition in cell proliferation was observed when chondrosarcoma cells were treated with 5 μM of AG1478 (Figure 5A), indicating that constitutive activation of EGFR sustains chondrosarcoma cell proliferation. In contrast, no effect of AG1478 on proliferation of SW1353 chondrosarcoma cells was observed either at 1 μM or at 5 μM (Figure 5A). We next investigated whether chondrosarcoma cell migration, that may influence metastatic phenotype, is affected following EGFR inhibition. To this end, we analyzed the effect of AG1478 on cell migration at 24 h and 48 h treatment using Oris™ cell migration assay. The results clearly show that treatment of chondrosarcoma cells with the EGFR inhibitor strongly reduced the migration of HEMC-SS cells compared to control cells (DMSO) (Figure 5B). Indeed, the population of cells that moved to detection zone at 24 h and 48 h of treatment is significantly reduced in AG1478-treated cells compared to vehicle-treated cells (control) as indicated by the size of the area closure (Figure 5B). However, no significant effect on the migration of SW1353 chondrosarcoma cells was observed at 24 h or 48 h of AG1478 treatment (Figure 5C). Altogether, these results support the notion that inhibition of EGFR in chondrosarcoma cells HEMC-SS decreased cell proliferation and migration.

### Inhibition of EGFR induces G0/G1 cycle arrest in chondrosarcoma cells

**Figure 6:**
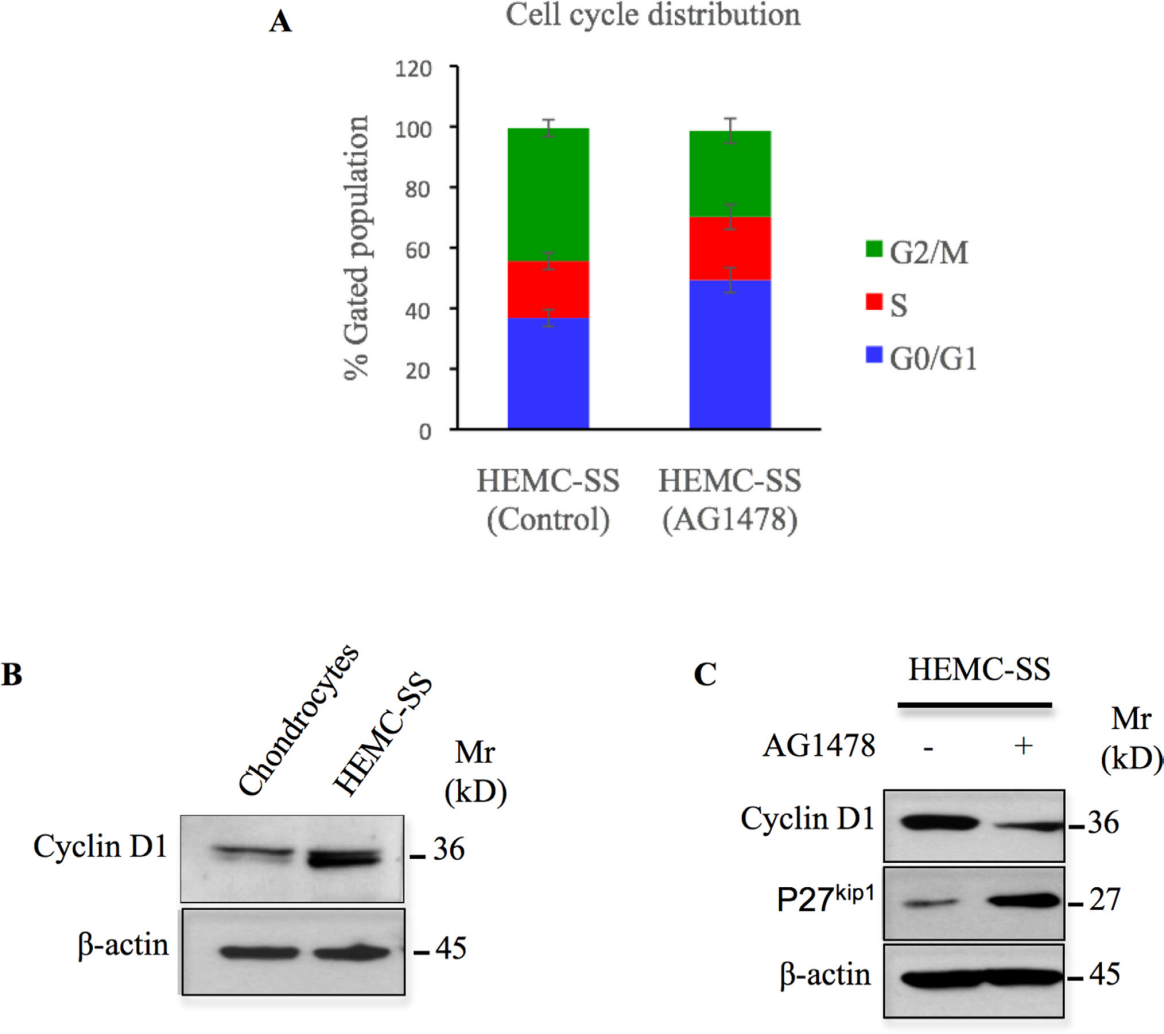
AG1478 produces cell cycle arrest, induces p27 and inhibits cyclin D1 in chondrosarcoma cells. (**A**) Cells were treated with 1 μM of AG1478 or with DMSO (control) for 24 h and cell cycle distribution was measured by Propidium Iodide flow cytometry. Abbreviations: M, mitosis; S, DNA duplication phase; G0/G1, gap between end of M-phase and start of S-phase; G2, gap between end of S-phase and start of M-phase. Data are expressed as mean (*n* = 3) ± SD, (^*^P < 0.05). (**B**) Western blot analysis of the expression of cyclin D1 in human primary chondrocytes and chondrosarcoma HEMC-SS cells. (**C**) Detection of cyclin D1 and p27 in chondrosacoma HEMC-SS treated with AG1478 (1 μM) or DMSO (control) for 24h. Data are representative of three independent experiments (*n* = 3).

To investigate the mechanism by which AG1478 mediates cell growth inhibition in HEMC-SS chondrosarcoma cells, we performed flow cytometry analysis to determine the effect of EGFR inhibition on cell cycle progression. Treatment of chondrosarcoma cells with AG1478 for 24 h resulted in the accumulation of cells in the G0/G1 phase and a concomitant depletion of cells in the G2/M phase (Figure 6A). The percentage of cells in the G0/G1 phase increased significantly from 36.8% to 49.3% while S-phase cells showed only a slight increase 18.8% to 20.9%. The percentage of G2/M-phase cells decreased from 43.9% in the control cells to 28.4% in the treated cells (Figure 6A). This finding indicates that cell cycle distribution was blocked in the G0/G1 phase when chondrosarcoma cells are treated with AG1478. This result, suggest that inhibition of EGFR causes growth arrest through G0/G1 cell cycle blockage.

To elucidate the molecular mechanism involved in the arrest of chondrosarcoma cells in G0/G1 phase by AG1478, we first investigated the expression of cyclin D1 that regulates G1 phase progression, in both chondrosarcoma cells and in human primary chondrocytes. We found that cyclin D1 is expressed at high amount in chondrosarcoma cells compared to primary chondrocytes, indicating that the expression of cyclin D1 is up-regulated in chondrosarcoma cells (Figure 6B). Interestingly, treatment of chondrosarcoma cells with AG1478 produced a strong decrease in the expression of cyclin D1 (Figure 6C). Given that progression through the G1 phase is achieved by the activity of cyclin-dependent kinases and is negatively regulated by cyclin-dependent kinases inhibitors (CKI), we analyzed the effect of AG1478 on the expression of the CKI, p27^kip1^. As shown in Figure 6C, treatment of chondrosarcoma cells with AG1478 strongly induces the expression of p27^kip1^. Altogether, these data indicate that AG1478-induced arrest of chondrosarcoma cells in G0/G1 phase involves downregulation of cyclin D1 expression and upregulation of p27^kip1^ tumor suppressor gene.

### Inhibition of EGFR induces apoptosis in chondrosarcoma

**Figure 7:**
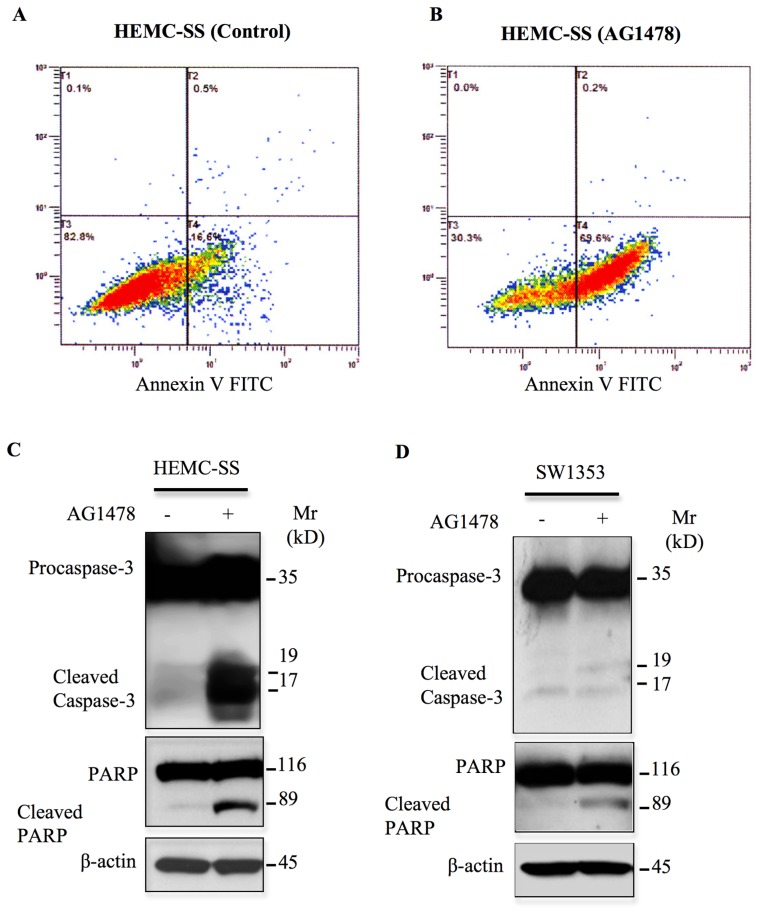
AG1478 induces apoptosis in chondrosarcoma cells. (**A**) Representative apoptosis profile of cells treated with DMSO (left panel) or (**B**) 1 μM of AG1478 (right panel) for 3 h stained with Annexin V-FITC Conjugate and Propidium Iodide (PI) using Alexa Fluor® 488 annexin V/Dead Cell Apoptosis kit and analyzed by flow cytometry. (**C**) and (**D**) HEMC-SS and SW1353 chondrosarcoma cells were treated with 1 μM of AG1478 or DMSO (control) overnight and assayed by western blot to assess the apoptotic markers Caspase-3 and PARP. Representative immunoblot shows the effect of AG1478 on the protein levels of Cleaved Caspase-3 and Cleaved PARP. β-actin was used as a loading control. Data are representative of three independent experiments (*n* = 3).

Next, we investigated whether AG1478 induces apoptosis in chondrosarcoma cells. To this end, cells were treated with AG1478 for 24 h and apoptosis was analyzed using Annexin V-FITC. The results reveal that AG1478 induces significant apoptosis (53%) in chondrosarcoma cells (Figure 7A and 7B). To confirm these observations we looked at the expression of key proteins involved in apoptotic pathway upon AG1478 treatment by western blotting. Treatment of cells with the AG1478 caused significant cleavage of Caspase-3 and PARP (Figure 7C), indicating that cells undergo apoptosis. These results reveal that inhibition of EGFR induces apoptosis in chondrosarcoma cells HEMC-SS, therefore indicating that the survival of these tumor cells rely strongly on constitutive activation of EGFR. In contrast, treatment of SW1353 chondrosarcoma cells with the EGFR inhibitor, AG1478 did not induce significant cleavage of both Caspase 3 and PARP (Figure 7D), suggesting that survival of these tumors may involve signaling pathways other than the EGFR pathway.

### EGFR inhibition down-regulates the expression of MMP-13 and MMP-3

**Figure 8:**
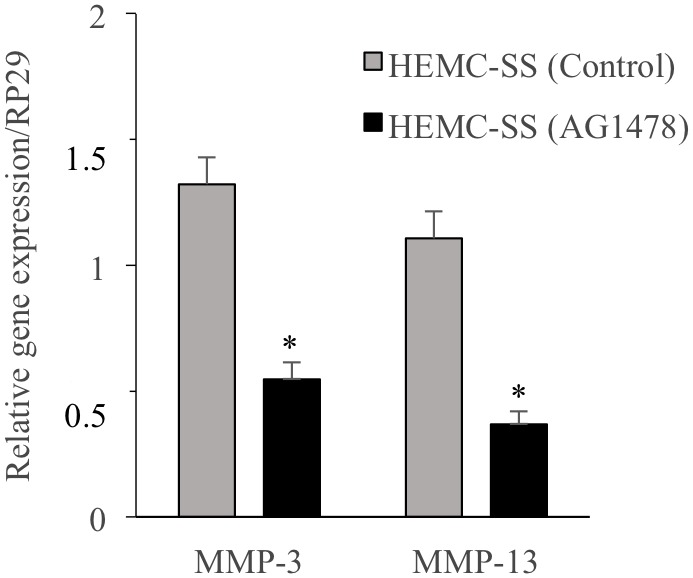
Inhibition of EGFR down-regulates the expression of the Matrix Metalloproteinases MMP3 and MMP13. Fold changes of MMP3 and MMP13 expression normalized to control in chondrosarcoma HEMC-SS cells treated with AG1478 (1 μM) or DMSO (vehicle) (*n* = 3). Data are representative of at least three experiments. Q-PCR values were normalized for the housekeeping gene ribosomal protein S29 and are expressed as relative expression compared with control. Data are expressed as mean ± S.D. Statistical analysis was performed with an unpaired Student’s *t*-test (^*^p < 0.05).

Matrix metalloproteinase-13 (MMP-13) also named collagenase-3 and MMP-3 named stromelysin-1 are of particular importance as they are responsible for the degradation of collagen and proteoglycans that are the major components of cartilage ECM, therefore they may play an important role in metastasis of chondrosarcoma cells. To investigate whether inhibition of EGFR affects the expression of MMP-13 and MMP-3, chondrosarcoma cells were treated with the inhibitor AG1478 and the expression of both genes was analyzed by qPCR. Interestingly, AG1478 reduces by about 47% and 68% the expression of MMP-13 and MMP-3, respectively (Figure 8). These results indicate that AG1478 decreases the expression of metastasis-related proteins in HEMC-SS chondrosarcoma cells and suggest that inhibition of EGFR may reduce chondrosarcoma metastasis.

### Constitutive EGFR signaling mediates aberrant activation of ERK1/2 and AKT in grade III chondrosarcoma cells

**Figure 9:**
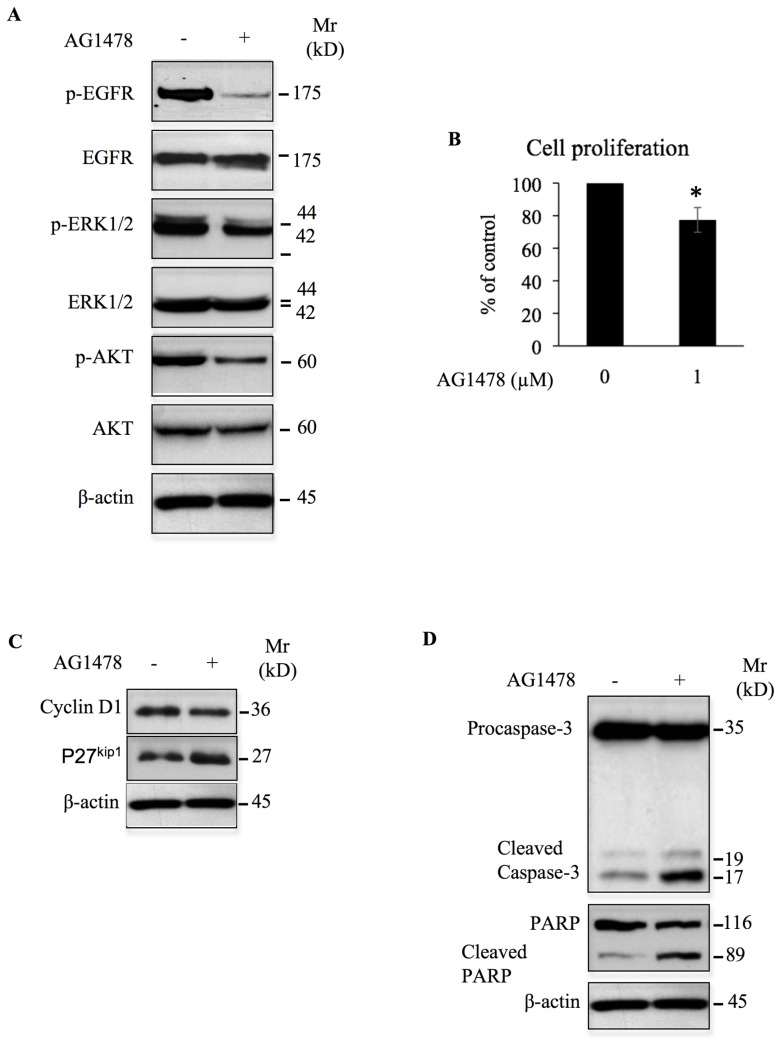
AG1478 inhibits oncogenic signals and cell growth, and induces apoptosis in high-grade chondrosarcoma cells. (**A**) Analysis of the effect of AG1478 (1 μM) on the phosphorylation of EGFR and on the activation of ERK1/2 and AKT downstream signaling pathways in chondrosarcoma cells CH2879. Control cells were treated with DMSO (vehicle). (**B**) Chondrosarcoma CH2879 cells were treated with DMSO (Control) or with 1 μM of AG1478 and cell proliferation was analyzed by Click-iT^®^ EdU as described in Material and Methods. Bar graph shows cell proliferation expressed as percentage relative to control cells. Data are expressed as mean (*n* = 3) ± SD. ^*^P < 0.05 versus control. (**C**) Detection of cyclin D1 and p27 in chondrosacoma CH2879 treated with AG1478 (1 μM) or DMSO (control) for 24 h. Data are representative of three independent experiments (*n* = 3). (**D**) Cells were treated with 1 μM of AG1478 or DMSO (control) overnight and assayed by western blot to assess the apoptotic markers Caspase-3 and PARP. b-actin was used as a loading control. Data are representative of three independent experiments (*n* = 3).

To determine whether EGFR activation contributes to progression to a higher grade lesion, we used chondrosarcoma cells, CH2879 that were established from grade III chondrosarcoma tumor of bone. Analysis of the phosphorylation status of EGFR indicated that the receptor is activated in CH2879 cells (Figure 9A), suggesting that EGFR receptor is aberrantly activated in these grade III chondrosarcoma cells. Treatment of the CH2879 chondrosarcoma cells with AG1478 strongly inhibits the phosphotylation of the EGFR receptor (Figure 9A). Interestingly, inhibition of EGFR by the TKI leads to significant reduction in the phosphorylation of both ERK1/2 and AKT (Figure 9A), indicating that the activation of oncogenic signals ERK1/2 and AKT in CH2879 cells rely on EGFR activation. Given the important role of ERK1/2 and AKT signaling pathways in cell proliferation and apoptosis, we hypothesize that inhibition of EGFR may affect CH2879 cell growth and survival. To test this hypothesis, we treated CH2879 cells with the EGFR inhibitor AG1478 for 24 h and analyzed cell proliferation and apoptosis. As shown in Figure 9B, up to 20% inhibition in cell proliferation was observed when chondrosarcoma cells were treated with 1 μM of AG1478, indicating that constitutive activation of EGFR sustains the proliferation of the grade III chondrosarcoma cells, CH2879. Interestingly, treatment of chondrosarcoma cells with AG1478 decreased the expression of cyclin D1 and induced that of the CKI, p27^kip1^ (Figure 9C), suggesting that AG1478 inhibits cell proliferation through dowregulation of cyclin D1 and upregulation of p27^kip1^. Next, we investigated whether AG1478 induces apoptosis in CH2879 cells. For this purpose, cells were treated with AG1478 for 24 h and the cleavage of Caspase 3 and PARP were analysed by western blot. Treatment with AG1478 induces significant cleavage of both Caspase 3 and PARP (Figure 9D), indicating that AG1478 induces apoptosis in CH2879 chondrosarcoma cells and suggest that activation of EGFR promote the survival of the tumor cells. Altogether, these results strongly support the notion that aberrant activation of EGFR is critical for chondrosarcoma tumor growth and survival and may contribute to progression to higher-grade lesion.

## DISCUSSION

In this study, we showed that EGFR is constitutively active in chondrosarcoma tumors of high-grade, whereas it is not active in low-grade tumors, suggesting that EGFR activation may be involved in chondrosarcoma tumor progression and therefore could be used as a biomarker to monitor the progression of chondrosarcoma tumors. Clinical trials have shown that inhibition of EGFR by TKIs such as gefitinib or antibodies such as cetuximab have proven effective in human non-small cell lung cancer [15, 16] and metastatic colorectal cancer [7, 17], respectively. In chondrosarcoma, gefitinib has been shown to reduce cell proliferation and migration of the cell lines, JJ2012 and SW1353 but the molecular mechanism involved has not been investigated [21]. However, we showed that SW1353 cells express high amount of EGFR but they do not produce EGF ligand and EGFR is not constitutively active in these cells. In addition, we found that EGFR inhibition with AG1478 did not affect the proliferation of SW1353 cells. Therefore, how gefitinib inhibits SW1353 cell proliferation remains to be elucidated.

Our study reveals that aberrant activation of oncogenic signals, ERK1/2 and AKT/mTOR is driven by constitutive activation of EGFR. Indeed, treatment of chondrosarcoma cells with AG1478 inhibits the phosphorylation of EGFR receptor and of ERK1/2 and AKT/mTOR. Chondrosarcoma tumors produce high amount of extracellular matrix proteins that may play a role in drug resistance. Three-dimensional cell culture models allow the synthesis and deposition of extracellular matrix and are more likely to mimic natural tumor microenvironment *in vitro.* These models become essential tools in cancer research, notably for testing the efficacy of anticancer drugs. Interestingly, we showed that AG1478 at a concentration of 5 μM is able to strongly inhibit the phosphorylation of EGFR and reduced the activation of down-stream pathways in chondrosarcoma cells cultured in 3D, indicating that chondrosarcoma did not show strong resistance to the drug in the 3D culture model used. Although other factors such as low vascularisation of chondrosarcoma tumors may play a role in the resistance of chondrosarchomas to chemotherapy, the 3D cultures results suggest that AG1478 may be effective in reducing tumor growth *in vivo*, however this has not been tested in this study.

Aberrant activation of oncogenic signals, ERK1/2 and AKT/mTOR in chondrosarcomas may sustain cell proliferation and survival. In line with this, it has been reported that treatment of rat chondrosarcoma cells with mTOR inhibitor, everolimus blocked cell proliferation and tumor progression [22]. Owing to the role of ERK1/2 and AKT/mTOR in cell proliferation and survival, and given that inhibition of EGFR strongly decreased the constitutive activation of these pathways, we investigated a potential effect of AG1478 on cell survival and proliferation. We found that the TKI induced apoptosis in chondrosarcoma cells as evidenced by activation of Caspase-3 and cleavage of PARP, indicating that EGFR activation is required for chondrosarcoma survival.

We showed that AG1478 inhibits the proliferation of chondrosarcoma cells. Given that ERK1/2 pathway has a pivotal role in facilitating cell proliferation and cycle progression by activating cell cycle regulatory proteins during G0/G1 [23], we analyzed whether the TKI impairs cell cycle progression. Our data revealed that inhibition of EGFR by AG1478 produced arrest in the G0/G1 phase, suggesting that AG1478 exerts growth-inhibitory effects in chondrosarcoma cells by causing G0/G1 cell cycle arrest probably through inhibition of ERK1/2 signaling.

Our data indicated that cyclin D1 is up-regulated in chondrosarcoma cells. Given that, overexpression of *cyclin D1* is correlated with tumor differentiation, poor survival, increased metastasis and resistance to certain cytotoxic drugs including tamoxifen [24–27] targeting cyclin D may be considered as an effective strategy for the treatment of chondrosarcoma. In line with this, the results obtained in the present study revealed that inhibition of EGFR activation with AG1478 induces inhibition of cyclin D1 expression. In addition, we showed that AG1478 treatment of chondrosarcoma cells strongly induced the expression of the cyclin/cdk inhibitor p27^kip1^. Of note, increased levels of p27^kip1^ protein typically cause cells to arrest in G1 phase by inhibiting the catalytic activity of cdks. In line with this, our results showed that treatment with AG1478 produced cell cycle arrest at G0/G1 phase. Interestingly, it has been reported that overexpression of EGFR in cancer cells plays a role in accelerated proteolysis of p27 protein and allow the cancer cells to undergo rapid division and uncontrolled proliferation [28]. Accordingly, inhibition of the EGF receptor with AG1478 increased the levels of p27^kip1^ protein in chondrosarcoma cells.

The metastatic process is complex, and tumor cells need to overcome many barriers to reach and grow in distant organs [29]. Chondrosarcoma metastasis needs degradation of ECM which is mainly composed of collagen and proteoglycans. Two main enzymes are involved in the degradation of ECM, MMP-13 which exhibits high activity towards collagen and MMP-3 that degrades proteoglycans [30]. It has been reported that chondrosarcoma produces high amount of MMP-13, suggesting that it may facilitate cartilage destruction through degradation of type II collagene and remodeling of collagenous ECM [31]. MMP-13 expression is also reported in primary and metastatic melanomas [32] and has been shown to enhance the invasion capacity of HT1080 fibrosarcoma cells [33]. Here, we showed that AG1478 treatment of HEMC-SS chondrosarcoma cells inhibits the expression of MMP-13 and MMP-3, suggesting that inhibition of EGFR may impair metastatic potential of chondrosarcoma. In line with this, it has been shown that EGFR activation in melanocytes resulted in a strong induction of MMP-13 in ERK1/2-dependent manner [34].

The use of therapeutic monoclonal antibodies in patients with solid tumors has been most successful with classes of antibodies targeting the ERBB family including EGFR [20, 35]. Here, we showed that EGFR neutralizing antibodies, directed against the external domain of the receptor, strongly inhibits the oncogenic signals, ERK1/2 and AKT triggered by EGFR activation. This indicates that monoclonal antibodies therapy may be beneficial for the treatment of chondrosarcoma with activated EGFR through a paracrine/autocrine loop. Taken together, our study reveals that EGFR is activated predominantly in high-grade chondrosarcoma tumors and may be used as a biomarker of chondrosarcoma cancer progression. Because of their heterogeneity, with differences in invasive and metastatic behaviour, it is important to identify biological markers that will allow for a more accurate estimation of prognosis in patients with these tumors. This study also reveals that constitutive EGFR activation drives the oncogenic signals in chondrosarcoma and suggests that inhibition of EGFR by TKIs or neutralizing antibodies may be proven effective in the treatment of high-grade chondrosarcoma.

## MATERIALS AND METHODS

### Cell culture and treatments

The human chondrosarcoma cell lines HEMC-SS (extraskeletal myxoid chondrosarcoma) and SW1353 (grade II chondrosarcoma of bone) were obtained from the European Collection of Animal Cell Cultures (ECACC; Salisbury, UK) and the American Type Culture Collection (ATCC; LGC, France), respectively. The human chondrosarcoma cell line CH2879 (grade III chondrosarcoma of bone) was obtained from Pr. Rosario Gil-Benso (The University of Valencia, Spain) [36]. They were cultured in Dulbecco’s modified Eagle’s medium/F12 (F12/DMEM) (1:1) supplemented with 10% (v/v) fetal bovine serum, penicillin (100 U/ml)/streptomycin (100 μg/ml), and 1 mM glutamine at 37° C in a humidified incubator containing 5% CO2. Chondrosarcoma cells were seeded in 6-well plates at 2 × 10^5^ cells per well, allowed to attach overnight then treated with the EGFR specific inhibitor, tyrphostin AG1478, (Sigma, Saint Louis MO) or vehicle (DMSO) at a concentration of 1 μM or 5 μM. Treatment of chondrosarcoma cells with neutralizing anti-EGFR antibody (Clone LA1, Millipore, Eschborn, Germany) or IgG (control) was performed overnight at a concentration of 5 μg/ml and 10 μg/ml, respectively. At the end of the treatment period, cells were washed with PBS and harvested for western blot and mRNA expression analyses.

Stimulation of chondrosarcoma cells SW1353 with conditioned medium from chondrosarcoma HEMC-SS cells was performed by replacing SW1353 chondrosarcoma cell medium with conditioned medium from HEMC-SS cells that were grown for 24 h. The conditioned medium was centrifuged at 5,000 × g for 10 min before addition to SW1353 cells for 1 h. Cells were then washed with PBS and collected for western blot.

### 3D culture of chondrosarcoma cells

HEMC-SS cells are cultured until 75% confluency in petri dishes then trypsinized and embedded in a 2% alginate solution in 0,09% NaCl (w/v) at 10^6^ cells/ml. Beads are formed by adding dropwise the alginate solution containing cells through a 18G syringe in a 102 mM CaCl_2_ solution. After 3 washes with 0,09% NaCl, beads are cultured in complete medium containing 1mM CaCl_2_ for 1 week. Beads are further cultured 3 days without Cacl_2_ then treated with DMSO (control) or with 1 μM or 5 μM of AG1478 for 24 h. Beads are then lysed in citrate/EDTA (50mM) (pH 6,8) buffer for 20 min, centrifuged 10 min at 300 × g, washed with PBS and lysed in RIPA buffer (150 mM NaCl, 50 mM Tris-HCl, pH 7.5, 1% deoxycholate, 0.1% SDS, 1% Triton X-100) supplemented with protease and phosphatase inhibitors (Roche Diagnostics, Indianapolis, IN, USA). Cell lysates were sonicated on ice and protein concentration of the samples was determined by the Bradford method.

### Chondrosarcoma tissue microarray and immunohistochemistry analysis

Chondrosarcoma tissue microarray, containing 27 cases of chondrosarcoma including well (grade I), moderately (grade II) and poorly (Grade III) differentiated was obtained from US Biomax (OS803, US Biomax, Inc, USA). Immunohistochemistry staining was performed on chondrosarcoma tissue microarray using the ImmPRESS™ HRP Universal Antibody Polymer Detection Kit (Vector Laboratories, CA, USA). Paraffin-embedded sections were deparaffinized in alcohol baths, then washed in water. Antigen unmasking was performed using HIER citrate buffer pH=6.0 (Zytomed, Germany) for 6 h at 70° C. Then, peroxidase activity is quenched for 10 min using Peroxid-Block buffer (Zytomed, Germany). Sections were subsequently blocked for 20 min with normal horse blocking buffer and incubated at 4° C overnight with primary antibodies, phospho-EGFR (p-EGFR, diluted 1:400, Cell signalling, Danvers, USA) or EGF (diluted 1:250, GeneTex, CA, USA) in antibody dilutent (Dako, CA, USA). Sections were washed once with PBS, incubated with ImmPRESS reagent for 30 min and washed twice with PBS before incubated with peroxidase substrate (Permanent AEC kit, Zytomed, Germany) until stains develop. Sections were then counterstained with hematoxylin, cleared and mounted. Immunohistochemistry images were obtained by using Leica DMD 108 microscope (Leica Microsystem, Germany).

### Small interfering RNA transfection

Chondrosarcoma cells were seeded in 6-well plates at 2 × 10^5^ cells per well and grown overnight at 37° C. Then, cells were transfected with 25 nM small interfering RNA (siRNAs) to human EGFR; siEGFR(1):CAGGAACTGGATATTCTGAAA, siEGFR(2):CC CATCCAATTTATCAAGGAA, siEGFR(3):TACGAA TATTAAACACTTCAA or control siRNA, (siControl, Scrambled, Qiagen, Hilden, Germany) using DharmaFECT transfection reagent (ThermoFisher Scientific, Waltham, USA) according to the manufacturer’s instructions. At 48 h post-transfection, cells were washed with PBS and harvested for immunoblotting to determine the change in the expression and the phosphorylation status of relative proteins.

### Western blot analysis

After treatment, cells were washed with ice-cold PBS and total protein was extracted using RIPA buffer (150 mM NaCl, 50 mM Tris-HCl, pH 7.5, 1% deoxycholate, 0.1% SDS, 1% Triton X-100) supplemented with protease and phosphatase inhibitors (Roche Diagnostics, Indianapolis, IN, USA). Cell lysates were sonicated on ice and protein concentration of the samples was determined by the Bradford method. Proteins from cell lysate or conditioned medium were separated on 10% SDS-PAGE gels, transferred to a PVDF membrane (BIO-RAD, CA, USA), and subsequently blocked in PBS-Tween 20 containing 5% nonfat milk for 1 h at room temperature. Membranes were incubated at 4° C overnight with primary antibodies, phospho-EGFR (Y1068), phospho-ERK1/2, ERK1/2, phospho-AKT, AKT, phospho-mTOR, mTOR, cyclin D1, p27, Caspase-3, or PARP (diluted at 1:1000, Cell signaling, Danvers, USA), EGFR Pan (diluted 1/10000 Epitomics, CA, USA), EGF (diluted at 1:500, GeneTex, CA, USA). β-actin antibodies (Sigma) were used at a dilution of 1:5000. The membranes were washed three times for 10 min each with TBST and incubated in horseradish peroxidase-conjugated rabbit or mouse secondary antibodies (1:2000, Cell signaling) for 1 h at room temperature. The blots were then developed using Clarity Western ECL substrate (BIO-RAD) according to the instructions of the manufacturer.

### Real-time quantitative PCR

Total RNA from cells was extracted using the RNeasy Mini Kit (Qiagen, Hilden, Germany) based on the manufacturer’s instructions. The reverse transcription was performed using 500 ng of total RNA from each sample with iScript Ready to use cDNA supermix (BIO-RAD). Quantitative PCR was performed with iTaq™ Universal SYBR Green Supermix kit (BIO-RAD) using StepOne Plus™ Real-Time PCR Systems (Applied Biosystems, CA, USA). Expression of MMP-13 and MMP-3 was measured using specific primers (Qiagen). PCR cycling parameters were 30 s at 95° C, 40 cycles of 30 s at 95° C and 60 s at 60° C. Expression levels of target genes were normalized to ribosomal protein S29 RNA level.

### Cell migration assay

The cell migration was measured using the Oris™ 96-well cell migration assay kit (Platypus Technologies, Madison, USA) following the manufacturer’s instructions. Briefly, on the first day, 5 × 10^4^ cells were seeded in each well. On day 2, the stopper was removed to allow cells to migrate into the detection zone. Culture medium containing AG1478 (5 μM) or DMSO (control) was added and cell migration to central zone was monitored at 24 h and 48 h. The picture of the cells that migrated into the detection zone was taken using inverted microscope Leica DMI3000 B (Leica Microsystems, Germany).

### Cell proliferation assay

The cell proliferation was measured by Click-iT^®^ EdU (ThermoFisher Scientific, Waltham, USA) assay, according to the manufacturer’s protocol. Cells were seeded in 96-well plates at 2 × 10^4^ cells per well. On day two, culture medium containing AG1478 or DMSO (control) was added for 24 h, then cells were exposed to 10 μM of 5-ethynyl-2′-deoxyuridine (EdU) for additional 4 h at 37 ° C, fixed with Click-iT^®^ EdU Fixative for 5 min at room temperature and incubated with 50 μL of Click-iT^®^ reaction cocktail for 25 min. Cells were then washed twice with 200 μL per well of Amplex^®^ UltraRed buffer and incubated with 100 μL per well of Amplex^®^UltraRed for 15 min at room temperature before addition of 10 μL per well Amplex^®^ UltraRed stop reagent. Cells were analyzed on a plate reader with filter sets appropriate for Amplex^®^ UltraRed (excitation/emission, 568/585 nm).

### Analysis of apoptotic cells

Apoptosis was measured by Alexa Fluor® 488 annexin V/Dead Cell Apoptosis kit (ThermoFisher Scientific, Waltham, USA), according to the manufacturer’s protocol. Briefly, after treatment with AG1478 (5 μM) or vehicle (DMSO) for 3 h, cells were harvested and resuspended in 100 μL annexin-binding buffer and stained with 5 μL annexin-V-FITC and 1 μL Propidium Iodide (PI) for 15 min at room temperature in the dark. Then, 400 μL annexin-binding buffer was added and cells were analyzed on EPICS ALTRAII flow cytometry (Beckman, USA) using Multi-cycle software (Phoenix Flow Systems, USA). Results were expressed as percentage of PI negative and annexin-V positive apoptotic cells. All experiments were performed in triplicate.

### Cell cycle analysis

Cells were treated with AG1478 inhibitor (5 μM) or vehicle (DMSO) for 3 h then washed twice with ice cold PBS, collected by trypsinization and centrifuged. The pellets were resuspended in 1 ml cold PBS and 2.5 ml absolute ethanol and incubated on ice for 15 min. After centrifugation, cells were stained with 500 μl propidium iodide (PI)-solution (50 μg/ml PI, 0.1 mg/ml RNase A, 0.05% Triton X-100) for 40 min at 37° C. Cells were analyzed on EPICS ALTRAII flow cytometry (Beckman, USA). Cell distribution in the different phases of the cell cycle was determined with Multi-cycle software (Phoenix Flow Systems, USA). Results were expressed as percentage. All experiments were performed in triplicate.

### Statistical analysis

The results are presented as mean ± SD of three independent experiments. Statistical differences between control and treated groups were evaluated using Student’s test. A two-sided *P*-value <0.05 was considered statistically significant for all analyses.

## Abbreviations

CKI: cyclin-dependent kinases inhibitors
ECM: extracellular matrix
EGFR: epidermal growth factor receptor
MMP-13: Matrix metalloproteinase-13
PI: Propidium Iodide
RTKs: receptor tyrosine kinases
siRNA: small interfering RNA
TKIs: tyrosine-kinase inhibitors
